# Single-molecule targeted therapy shrinks lung lesions and improves bone metastases: A case report

**DOI:** 10.1097/MD.0000000000038874

**Published:** 2024-07-19

**Authors:** Jun Wei, Bei Hu, Huang Fang, Fangqi Zhang, Peng Wang

**Affiliations:** a Department of Digestive Diseases, Wuhan Jinyintan Hospital, Tongji Medical College of Huazhong University of Science and Technology, Hubei Clinical Research Center Diseases, Wuhan Research Center for Communicable Disease Diagnosis and Treatment, Chinese Academy of Medical Sciences, Joint Laboratory Infectious of Diseases and Health, Wuhan Institute of Virology and Wuhan Jinyintan Hospital, Chinese Academy of Sciences, Wuhan, People’s Republic of China; b Department of Radiology, General Hospital of Central Theater Command of the People’s Liberation Army, Wuhan, People’s Republic of China; c Department of Neurology, General Hospital of Central Theater Command of the People’s Liberation Army, Wuhan, People’s Republic of China; d Department of Pulmonary and Critical Care Medicine, The 987th Hospital of Joint Logistics Support Force of People’s Liberation Army, Baoji, People’s Republic of China; e Department of Pharmacy, The 987th Hospital of Joint Logistics Support Force of People’s Liberation Army, Baoji, People’s Republic of China.

**Keywords:** bone metastases, case report, lung adenocarcinoma, molecular targeted therapy, non-small cell lung cancer, Osimertinib

## Abstract

**Rationale:**

Bone metastasis is a common metastatic mode of advanced lung cancer and poses a great threat to the survival and quality of life of patients with this disease. However, the available literature has limited treatment options for advanced lung cancer with bone metastases.

**Patients concerns:**

A 76-year-old married male patient was underwent CT due to cough and sputum for 1 month. On CT, space-occupying lesions were found in the left inferior lobe of the lung, as well as multiple bone metastases in the vertebral body and ilium.

**Diagnoses:**

Pathologic sectioning of the lung lesion after puncture revealed invasive lung adenocarcinoma, and a genetic test revealed EGFR exon 21: L858R (64.60%).

**Interventions:**

Considering that the disease was not suitable for radiotherapy (extensive metastasis) and could not be treated with chemotherapy (poor underlying condition), the patient was given molecularly targeted therapy with osimertinib.

**Outcomes:**

After 10 months of standard treatment (80 mg orally, once a day), the lung lesions of the patients became significantly smaller, and the bone metastases distinctly improved. And the patient’s condition has not shown any signs of rebound with the one-year follow-up.

**Lessons subsections:**

In the present case, the bone metastases from lung adenocarcinoma almost completely disappeared after treatment with a single molecular targeted therapy agent, increasing the confidence in the treatment of advanced lung cancer.

## 1. Introduction

Bone is the most common metastatic target organ for patients with lung cancer, especially non-small cell lung cancer (NSCLC).^[1]^ Previous literature has shown that bone metastases occur in approximately 30% to 40% of lung cancers.^[2]^ The prognosis is very poor for lung cancer patients with bone metastasis. Unfortunately, for the majority of lung cancer patients with bone metastases, it is difficult to achieve clinical remission during treatment.^[3]^ Molecular targeted therapy offers the possibility of revolutionizing cancer theranostics in the new era of precision oncology.^[1]^ Thus, can molecular targeted therapy also achieve good results in patients with bone metastatic lung cancer? This needs clinical data to prove. However, there are few previous studies in this area. Therefore, we report a patient with multiple bone metastases from lung cancer, and multiple bone metastases were distinctly improved with single-molecule targeted therapy.

## 2. Case report

**Figure 1.**
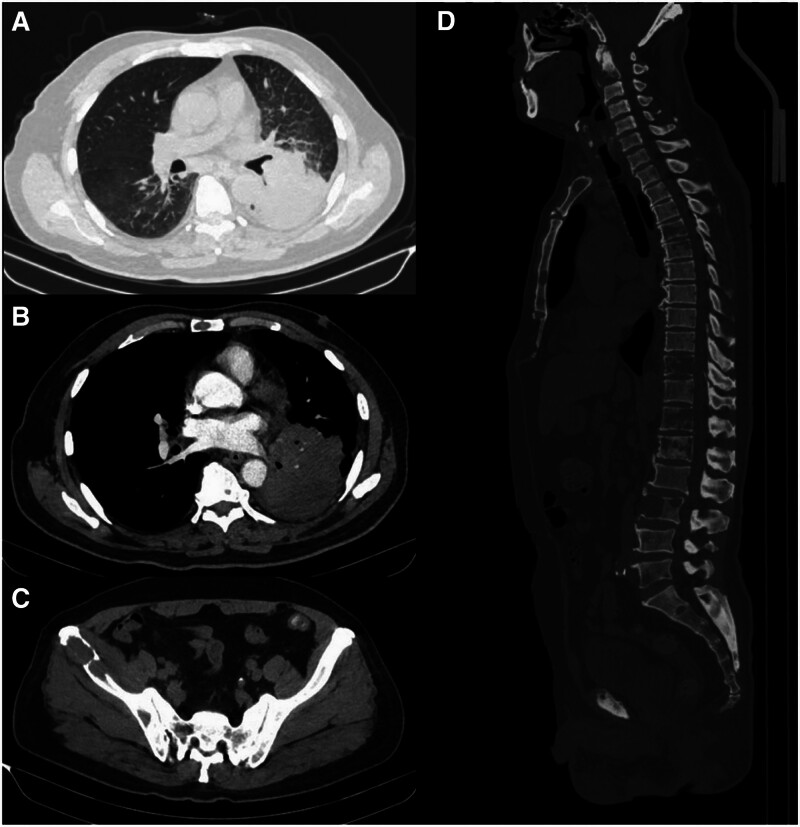
A 16-slice computed tomography (CT) scan revealed a space-occupying lesion with obstructive pneumonia in the lower lobe of the left lung (9.8 × 6.9 cm) and osteolytic destruction. (A) The space-occupying lesion in the lung window; (B) the space-occupying lesion in the mediation window; (C) osteolytic destruction of the right ilium; (D) osteolytic destruction of the vertebral body.

A 76-year-old married male patient was hospitalized for cough and sputum for 1 month. One month before admission, the patient had no cough or sputum, white mucus sputum, approximately 10 mL per day, or blood. He had no chest pain, fever, chest tightness, or choking. His weight loss was approximately 8 kg in that month. During medical examination, his armpit temperature was 36.5 °C, his pulse rate was 110 breaths/min, his breathing rate was 20 breaths/min, and his blood pressure was 155/90 mm Hg. He was conscious and exhibited stable breathing, no obvious swelling of superficial lymph nodes, a percussive sound in either lung, low respiratory sounds in the lower lobe of the left lung and scattered wet rales. He was auscultated, and clear respiratory sounds in the right lung and no dry or wet rales were auscultated. His heart rate was 110 beats/min, and she had sinus rhythm and cardiac auscultation but no murmur. The abdomen was flat and soft, there was no tenderness, rebound pain or muscle tension in the whole abdomen, the liver, spleen, and ribs were not palpated, there was suspicious spinal tenderness, and the lower limbs were not edematous. A computed tomographic scan revealed space-occupying lesions in the lower lobe of the left lung with obstructive pneumonia, and multiple lymph nodes with partial enlargement in the hilar area and mediastinum were found; these lesions were considered metastatic tumors. There was a small amount of fluid in the left thoracic cavity. Moreover, multiple osteolytic lesions in the ribs, vertebral body, and ilium were also found via computed tomographic scan; these lesions were considered multiple bone metastases by a radiologist (Fig. 1).

**Figure 2.**
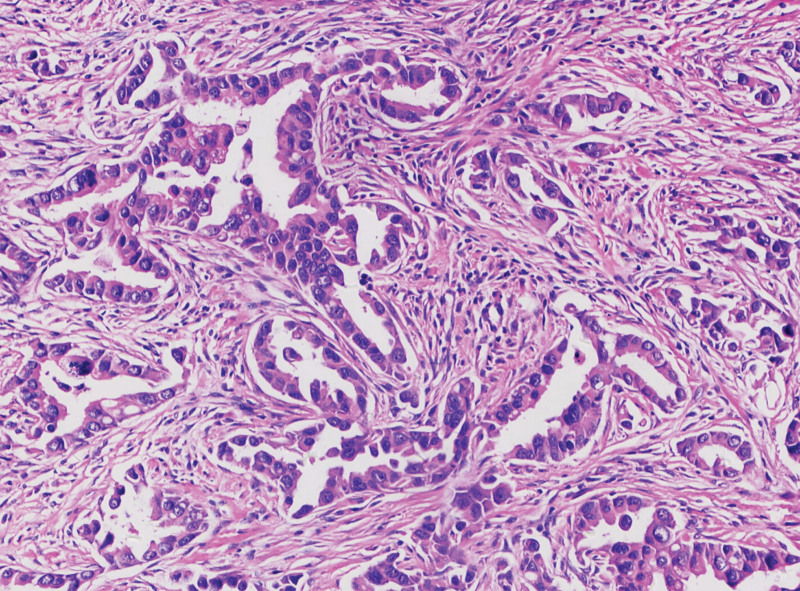
Histologic diagnosis via hematoxylin and eosin staining (original ×200) revealed invasive lung adenocarcinoma.

Blood routine and biochemical examinations showed no obvious abnormalities. The serum tumor marker level of the carcinoembryonic antigen was 195.79 ng/mL, the cytokeratin 19 fragment was 19.31 ng/mL, and the neuron-specific eosinophil level was 20.26 ng/mL. According to the current clinical data, patients are more likely to be diagnosed with unresectable advanced lung cancer. To obtain an accurate diagnosis, the patient underwent a puncture biopsy of the lung mass. Pathology of the patient suggested infiltrating adenocarcinoma of the left lung puncture tissue (Fig. 2). EGFR exon 21: L858R (64.60%) was detected via the use of puncture tissue. He was diagnosed with lung adenocarcinoma cT4N3M1c stage IVb and was EGFR-positive (according to the eighth edition TNM classification, innumerable nodules in the ipsilateral lobes were categorized as T4, and those in a contralateral lobe while multiple metastases in distant organs were M1, bilateral hilar and mediastinal lymphadenopathy were N3).

**Figure 3.**
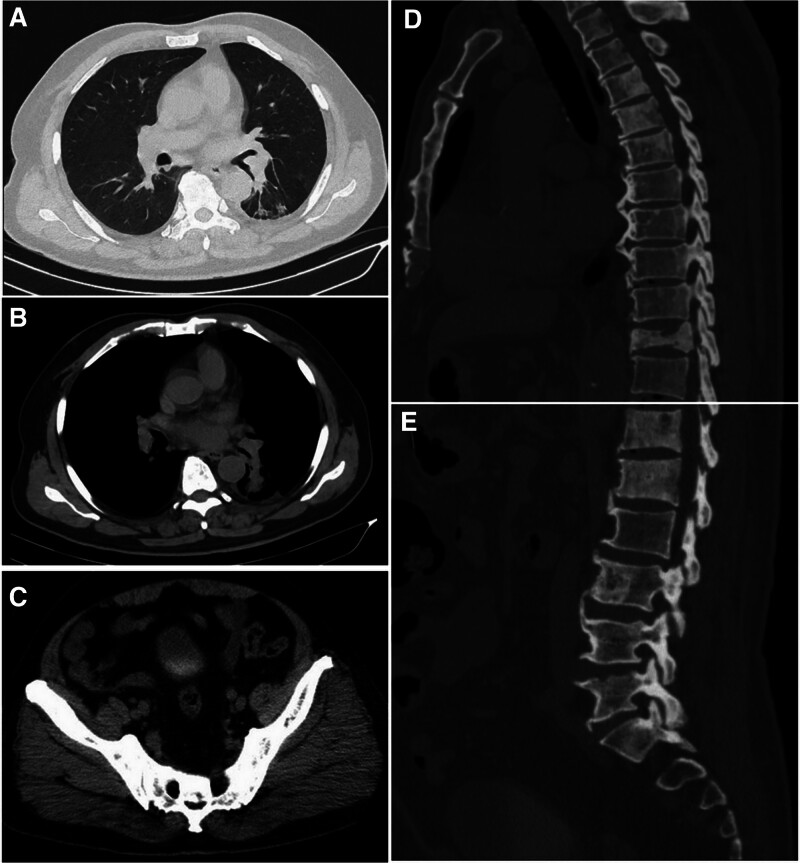
A 16-slice computed tomographic scan revealed that the left lung space-occupying lesion with obstructive pneumonia was almost completely absorbed, and the osteolytic destruction had disappeared. (A) Lung window; (B) mediation window; (C) right ilium; (D) thoracic vertebra; (E) lumbar vertebra.

Given the disease’s inability to respond to radiotherapy due to extensive metastasis and the patient’s inability to tolerate chemotherapy due to a poor underlying condition, a multidisciplinary team (MDT) opted for molecularly targeted therapy with osimertinib. After a 10-month course of standard treatment (80 mg orally, once daily), a reexamination CT scan revealed that the lung lesions were almost absorbed and that the bone metastases were distinctly improved (Fig. 3). And the patient’s condition has not shown any signs of rebound with the 1-year follow-up.

## 3. Discussion

Currently, early lung cancer is successfully treated using minimally invasive surgery.^[4]^ However, advanced lung cancer is one of the main causes of cancer death due to its aggressive nature and poor prognosis.^[5]^ Bone is one of the most common sites of metastasis in advanced lung cancer, and approximately 30% to 40% of lung cancer patients have bone metastasis.^[6]^ The survival time of lung cancer patients with bone metastasis is significantly shorter than that of patients without bone metastasis, and bone metastasis often causes severe clinical symptoms such as pain, pathological fracture and neurological impairment.^[7]^ With the progress of comprehensive treatment, patients with lung cancer bone metastasis are still difficult to treat clinically.

The most common treatment for bone metastases in patients with lung cancer is surgery. These types of surgeries can be divided into palliative symptom relief surgery and radical surgery. Although some early studies have suggested that surgical treatment could help improve the survival time and quality of life of lung cancer patients with bone metastasis, the latest literature has shown that surgery cannot significantly improve the survival time of patients and may also lead to different degrees of complications.^[8,9]^

Local palliative radiotherapy is also an important treatment for bone metastases of lung cancer and can effectively relieve patients’ pain and preserve bone function and integrity. However, the current literature suggests that this treatment does not extend 5-year survival but rather the length of skeletal-related events.^[10,11]^ Of course, local palliative radiation therapy may increase the incidence of lung, skin, hematopoietic and other related side effects, but it is manageable.^[11]^

Palliative supportive systemic chemotherapy is the standard treatment for patients with bone metastases in lung cancer and can effectively prolong the 5-year survival time.^[12]^ However, according to the published literature, there are no reports on the disappearance of lung cancer bone metastases caused by chemotherapy.

Immunotherapy is a treatment method for advanced lung cancer that has attracted much attention in recent years.^[13]^ It has shown great clinical benefit in the treatment of patients with lung cancer and is now used as a first-line treatment option for metastatic disease. The literature shows that the effectiveness of immunotherapy at different metastatic sites is significantly different.^[14]^ For example, after treatment with nivolumab, the response rate in the lymph nodes was significantly greater than that in other organs, such as the liver, adrenal glands and bones.^[15]^ Similarly, we did not find evidence in the literature that immunotherapy resulted in the disappearance of lung cancer bone metastases.

With the increase in understanding of the mechanism of bone metastasis-related cancer, the application of the combination of bisphosphonates (i.e., zoledronic acid) and receptor activator of nuclear factor kappa B ligand (RANKL) inhibitors (denosumab) in lung cancer patients with bone metastasis is also increasing.^[16]^ Bisphosphonate can inhibit the activity of osteoclasts and reduce the dissolution and destruction of bone trabeculae, thus preventing various skeletal-related events caused by tumor metastasis.^[17]^ However, only a few patients receive these drugs because they can increase the quality of life and economic burden. Finally, doctors are afraid of the possibility of osteonecrosis of the jawbone, which is one of the most serious adverse reactions.^[18]^

Targeted therapy has been established as the standard first-line treatment for patients with advanced NSCLC with epidermal growth factor receptor (EGFR) mutations.^[19]^ These drugs inhibit osteoclast activation by inhibiting tumor growth at bone metastases, the production of osteolytic factors in tumor cells, the proliferation of osteoblasts/stromal cells, and the differentiation of bone marrow cells into osteoclasts.^[20]^ Among the 4 types of bone metastasis, osteolytic, osteoblastic, osteoporotic, and mixed, osteolytic bone metastasis is more prevalent in NSCLC patients.^[21,22]^ Like in other malignancies, bone destruction due to bone metastases is caused by lung cancer cells disrupting the normally finely controlled balance between bone resorption and bone formation, leading to destruction and dissolution of bone tissue.^[23,24]^ In vivo studies have shown that EGFR is essential for osteoblast proliferation, and downregulation of EGFR signaling favors osteoprogenitor senescence in cortical bone and a decrease in bone formation.^[25,26]^ In addition to regressing EGFR-mutant NSCLC tumors in the bones, osimertinib could remodel the bones, as shown in another study.^[27]^ On the basis of the results of previous studies, molecular targeted therapy is also recognized as a possible effective way to improve bone metastasis in NSCLC patients.^[28]^ However, the literature has not revealed the exact mechanism of the disappearance of bone metastases after treatment of lung cancer bone metastases with molecular targeted drugs.

Finally, other interventional techniques, including spinal injections, nerve blocks, ablation (cryo-, thermo, or radiofrequency), and vertebroplasties, are available for lung cancer patients with bone metastasis, and previous research has shown good efficacy in pain control via these techniques.^[29–31]^

As a case report, this study has some limitations also. First, the experience of treating a single case is not strong enough to make a generalization. Second, this case was only followed up for 1 year, the long-term effect still needs further follow-up.

In conclusion, in the current era of studies on the specific mechanisms of lung cancer development, the number of advanced lung cancer patients who benefit from surgical treatment is limited. This case of individualized therapy suggested that molecular targeted therapy may be an effective treatment for lung cancer patients with bone metastasis from lung cancer via gene expression.

## Author contributions

**Data curation:** Jun Wei, Fangqi Zhang, Peng Wang.

**Formal analysis:** Jun Wei, Fangqi Zhang, Huang Fang, Peng Wang.

**Writing – original draft:** Jun Wei, Fangqi Zhang.

**Funding acquisition:** Bei Hu, Fangqi Zhang, Huang Fang, Peng Wang.

**Methodology:** Bei Hu, Huang Fang, Peng Wang.

**Writing – review & editing:** Bei Hu, Huang Fang, Peng Wang.

**Software:** Fangqi Zhang.

**Supervision:** Fangqi Zhang, Huang Fang, Peng Wang.

**Investigation:** Huang Fang, Peng Wang.

**Project administration:** Huang Fang, Peng Wang.

**Resources:** Huang Fang, Peng Wang.

**Conceptualization:** Peng Wang.
